# Groundwater monitoring for 1,3‐dichloropropene in high fumigant use areas of North America and Europe

**DOI:** 10.1002/ps.5398

**Published:** 2019-04-01

**Authors:** Ian J van Wesenbeeck, Steve Knowles

**Affiliations:** ^1^ Ilahe Environmental, LLC Salem OR USA; ^2^ Independent Environmental Consultant Newbury UK

**Keywords:** 1,3‐dichloropropene, groundwater, fumigant, monitoring

## Abstract

**BACKGROUND:**

1,3‐Dichloropropene (1,3‐D) is a soil fumigant used for the control of nematodes in high‐value fruit, nut and vegetable crops globally. Extensive water monitoring efforts have been undertaken over the past four decades by public and private institutions, given the widespread agricultural use of 1,3‐D, and environmental fate and metabolism data suggesting the potential for 1,3‐D to leach into groundwater. The aim of this study is to review the results of groundwater monitoring studies for 1,3‐D conducted in North America and the European Union (EU) since 1980.

**RESULTS:**

Analysis of > 50 000 water samples by state and federal agencies in the USA resulted in 151 detections of 1,3‐D. An additional 4000 samples analyzed in groundwater studies specifically targeting high 1,3‐D use areas in Europe and the USA resulted in 74 detections of 1,3‐D or its primary metabolites. The combined detection rate of 1,3‐D and its primary metabolites in high‐use areas of the EU and North America was 0.7%.

**CONCLUSIONS:**

The availability of extensive groundwater monitoring information developed through decades of study supports the conclusion that soil fumigation with 1,3‐D poses an inconsequential risk for drinking water exposure. The lack of significant detections of 1,3‐D from targeted monitoring studies is due to the high volatility of 1,3‐D, the rapid degradation of 1,3‐D in most agricultural soils, the rapid degradation of 1,3‐D and its metabolites in aerobic aquatic systems, and the rapid hydrolysis of 1,3‐D in water. © 2019 The Authors. Pest Management Science published by John Wiley & Sons Ltd on behalf of Society of Chemical Industry.

## 1 INTRODUCTION

1,3‐Dichloropropene (1,3‐D) is an essential pre‐plant soil fumigant used on a wide variety of fruit, vegetable, field, tree and vine crops, across widely diverse agricultural areas of the USA and Europe. It is used to protect billions of dollars' worth of crops globally from nematodes and other soil‐borne plant pests. 1,3‐D was introduced in 1955, and registered for use as a soil fumigant for agricultural use in the USA and European Union (EU) in 1966. Products containing 1,3‐D were re‐registration in the USA in 1998,^1^ and are currently undergoing the next scheduled round of registration review by the US Environmental Protection Agency (EPA), expected to be completed in 2021. The active substance 1,3‐dichloropropene was evaluated in the EU in 2009 but did not obtain approval due to new data requirements applicable to manufacturing impurities; however, a new dossier has been submitted and is currently under review to obtain European approval under EC Regulation 1107/2009 for uses as a nematicide in fruiting vegetable production, in open fields and in glasshouses.

Fumigating soils with 1,3‐D is an expensive, prescriptive treatment and growers apply 1,3‐D sparingly and selectively. Crop history and the known presence of nematodes and disease facilitate identification of appropriate application requirements. The labeled broadcast application rate for vegetable crops is ∼ 100–130 kg ha^−1^ for mineral soils and 275 kg ha^−1^ for muck or peat soils. The application rate for tree and vine crops is ∼ 380 kg ha^−1^, with applications typically made only at the time of orchard or vineyard planting, and hence occurring once every 20–30 years. The phase‐out of methyl bromide due to its ozone depletion potential, under the Montreal Protocol in 1987, has resulted in increased use of 1,3‐D which by 2011, had increased to 12 300 000 kg applied to 126 000 ha in the USA, making it the most applied soil fumigant in the country.^2^ 1,3‐D is applied as a pre‐planting soil fumigant that can be shank‐injected directly into the soil or applied by drip irrigation. 1,3‐D is injected into the soil at a depth of 0.3–0.46 m after which the soil is sealed by disking and rolling to minimize evaporation from the surface. In some cases, additional mitigation measures are applied to minimize the emission of fumigant from the soil surface, such as plastic film^3^ or reactive soil amendments such as ammonium thiosufhate.^4^ 1,3‐D can be applied by drip irrigation at depths of 0.01–0.15 m below the soil surface; however, plastic film may be required to minimize emission to the atmosphere.

Most 1,3‐D in the USA is applied as pure 1,3‐D (e.g. Telone II^5^); however, some 1,3‐D products include mixtures with chloropicrin to increase the spectrum of disease management. For example, Telone C‐17^6^ or C‐35 contain nominally 17% or 35% chloropicrin, respectively, whereas Pic‐Clor 60^7^ nominally includes 60% chloropicrin. 1,3‐D products are also formulated as emulsifiable concentrates (ECs) for application with drip irrigation systems, including In‐Line^8^ and Pic‐Clor 60 EC,^9^ nominally 33% and 60% chloropicrin, respectively.

1,3‐D consists of approximately equal proportions of *cis‐* and *trans‐*isomers. Both isomers of 1,3‐D degrade rapidly in soil and water into the *cis‐* and *trans‐*isomers of its primary metabolites, 3‐chloroallyl alcohol and 3‐chloroacrylic acid. 1,3‐D degrades via hydrolysis with a half‐life of 11 days in sterile water at pH 7 and 20 °C, whereas the aerobic soil metabolism half‐life ranges from 2 to 17 days at 20 °C.^10^ Owing to the vapor pressure of 1,3‐D (3700 Pa at 20 °C), volatilization to air is also a major route of dissipation, with ∼ 25–30% evaporating from soil within 2 weeks after shank‐injection into soil^11^ or after drip irrigation.^12^ The environmental fate of 1,3‐D has been reviewed in detail by Ruzo^10^ and Terry *et al.* 13 The low (Koc) suggests that it is mobile in soil, and has the potential to leach to groundwater. Numerous groundwater samples have been collected and analyzed for 1,3‐D, by government agencies starting in the 1980s, and since 1999, targeted groundwater monitoring studies have been conducted in areas of high fumigant use in the USA and EU. The EPA Office of Water (OW) has set a health advisory level (HAL) of 0.2 μg L^−1^ for 1,3‐D in drinking water, while the California Department of Pesticide Regulation (CDPR) has set a maximum contaminant level (MCL) of 0.5 μg L^−1^ and a public health goal of 0.2 μg L^−1^.

The purpose of this report is to review and summarize the extensive body of groundwater monitoring data that has been generated for 1,3‐D and its metabolites since the 1980s and is available in the public domain, as well as tap water (drinking water) and groundwater studies conducted by Dow AgroSciences.

## 2 METHODS

Public peer‐reviewed and published literature related to 1,3‐D groundwater monitoring was searched using the Google Scholar® and SciFinder® search engines, and proprietary 1,3‐D monitoring data generated by Dow AgroSciences LLC was obtained from internal study reports. The reported limit of detection (LOD) and/or limit of quantification (LOQ) for the analytes and matrices measured in the reported studies reflect the advances in analytical methodology over time.

## 3 RESULTS

### 3.1 United States federal and state agency groundwater monitoring 1980–1991

The US EPA sampled ∼ 1300 Community Water System (CWS) wells between 1988 and 1990 in the National Survey of Pesticides in Ground Water.^14^ That survey indicated that of the 1292 well water samples analyzed, there were no detections of 1,3‐D with a reporting limit of 0.05 μg L^−1^. Additionally, California and Florida, states with very high 1,3‐D demand and use, conducted monitoring programs in the late 1980s and early 1990s, and no detections of 1,3‐D were observed in California, although two detections occurred in Florida. A summary of the results of the EPA and state monitoring programs with respect to confirmed detections of 1,3‐D are shown in Table 1.

**Table 1:** Groundwater monitoring for 1,3‐dichloropropene in the USA prior to 1991

Program	Years	No. of samples	LOD (μg L^−1^)	No. of detections
EPA National Pesticide Survey	1988–1990	1292	0.05	0^a^
California	1985‐1990	20 394	variable	0
Florida	1983–1991	15 109	0.14	2^b^

LOD, limit of detection.

a Reporting limit 0.05 μg L^−1^.

b Stauffer, 1991 (pers. commun.) two detections were 0.28 and 7.83 μg L^−1^, with a reporting limit of 0.02 μg L^−1^.

The CDPR review of 1,3‐D concluded that it does not meet the physicochemical criteria for inclusion on the CDPR's groundwater protection list and had low potential to contaminate groundwater,^15^ which corroborates the conclusions of the World Health Organization evaluation of 1,3‐D.^16^ CDPR^15^ also reviewed targeted groundwater monitoring data specifically from sites where 1,3‐D had been applied that resulted in no detections of 1,3‐D. Further, in a study of groundwater in high fumigant use areas in California (Merced and Fresno counties) Maddy *et al.* 17 found no detections of 1,3‐D or 3‐chloroallyl alcohol above the LOD of 0.5 μg L^−1^.

### 3.2 Water quality portal

Groundwater and surface water samples collected and analyzed by state and federal government agencies including the EPA and United States Geological Survey were searched using the Water Quality Portal (WQP) managed by the National Water Quality Monitoring Council (http://www.waterqualitydata.us/). In total 11 700 ground and surface water samples from around the USA have been analyzed for 1,3‐D which was detected in 149 samples (1.27%) and the 99th percentile concentration was 0.25 μg L^−1^. Of the 11 700 samples analyzed for 1,3‐D, 6225 used an analytical method with LOD < 0.05 μg L^−1^, 1075 used a method with 0.05 < LOD < 1 μg L^−1^, 4043 used a method with 1 < LOD < 3 μg L^−1^ and 357 samples used a method with LOD ranging from 5 to 1000 μg L^−1^.

### 3.3 United States tap water monitoring study

Dow AgroSciences, LLC conducted a tap water (drinking water) monitoring study in five major 1,3‐D use regions in the USA as a condition of the re‐registration of 1,3‐D in the USA. In total, 503 monitoring sites were selected in high 1,3‐D fumigant use areas in the Upper Snake River basin (113 wells), the Central Columbia Plateau (82 wells), the North Platte River basin (108 wells), the Albemarle‐Pamlico Sound basin (90 wells), and the Georgia–Florida drainage basin (110 wells). Private homeowner wells were selected in unconfined aquifers adjacent to 1,3‐D fumigated fields with a minimum of 10 years of documented fumigant use history and were sampled four times between 19 April 2000 and 8 April 2001.

The majority of the monitored wells in Georgia, Florida, North Carolina and Idaho were located in shallow unconfined aquifers < 30 m deep. The Central Columbia Plateau study area in Washington State had ∼ 50% of the wells between 30 and 200 m deep, reflecting the deeper groundwater in that region. Approximately 50% of the wells in each region were between 30.5 and 91.5 m from the edge of a 1,3‐D treated field. Approximately once every 3 months for 1 year, from April 2000 to April 2001, tap water samples were collected in 20 mL volatile organic carbon vials before any filtration or treatment device, and were immediately frozen and shipped to the Dow AgroSciences analytical laboratory in Indianapolis, Indiana for analysis of 1,3‐D, 3‐chloroallyl alcohol and 3‐chloroacrylic acid by gas chromatography/mass spectrometry. LOD values were 0.015, 0.023, and 0.023 μg L^−1^, and LOQ values were 0.05, 0.092, and 0.046 μg L^−1^, for 1,3‐D, 3‐chloroacrylic acid and 3‐chloroallyl alcohol, respectively.

This tap water monitoring study showed that of the 1927 samples collected and analyzed for 1,3‐D and its 3‐chloroacrylic acid and 3‐chloroallyl alcohol metabolites (5781 total analyses), there were a total of two quantifiable detections of 1,3‐D, both < 0.15 μg L^−1^, and two detections that were less than the LOQ (0.05 μg L^−1^). There were a total of ten detections of the 3‐chloroallyl alcohol metabolite, all < LOQ, and 50 detections of the 3‐chloroacrylic acid metabolite (28 detections were < LOQ), with a maximum concentration of 0.14 μg L^−1^ (Table 2). Detections in any given well were transient, and no well showed a detectable level of any of the analytes for two consecutive sampling events. For the entire monitoring program, the detection rate was 0.2% for 1,3‐D, 0.5% for 3‐chloroallyl alcohol and 2.5% for 3‐chloroacrylic acid.

**Table 2:** Dow AgroSciences tap water monitoring study of 1,3‐dichloropropene use in five regions of the USA for 2000–2001

		No. of detections					
		1,3‐D^a^		Alcohol^b^		Acid^c^	
Study unit	No. of wells	>LOQ	trace^d^	>LOQ	trace	>LOQ	trace
Upper Snake River Basin (ID)	113	0	0	0	3	0	0
North Platte River Basin (NE)	108	0	0	0	0	0	0
GA/FL Coastal Plain	110	0	0	0	3	0	0
Central Columbia Plateau (WA)	82	2	2	0	4	6	7
Albermarle‐Pamlico Sound (NC)	90	0	0	0	0	16	21

a 1,3‐Dichloropropene LOD = 0.015 μg L^−1^, LOQ = 0.05 μg L^−1^.

b 3‐Chloroacrylic acid LOD = 0.023 μg L^−1^, LOQ = 0.05 μg L^−1^.

c 3‐Chloroallyl alcohol LOD = 0.023 μg L^−1^, LOQ = 0.09 μg L^−1^.

LOQ, limit of quantification; LOD, limit of detection.

d Trace concentrations are > LOD and < LOQ.

This tap water monitoring study was intended to be retrospective in nature and therefore wells in areas with significant 1,3‐D use (historically and at the time of the study) were selected for sampling. The most vulnerable wells, selected using rigorous vulnerability criteria in terms of depth to groundwater, soil type and proximity to treated fields, that could be found in each 1,3‐D use area were sampled. Therefore, the results of this tap water study represent a realistic and high‐quality data set for assessing exposure and risk to individuals consuming drinking water obtained from groundwater in 1,3‐D use areas in the USA. The results of this tap water study further corroborate the results of numerous state groundwater monitoring programs that tested for 1,3‐D in the USA since 1980, showing that even in high intensity 1,3‐D use areas, groundwater resources are not significantly contaminated by 1,3‐D or its metabolites.

### 3.4 Golf course groundwater monitoring study in Florida

Dow AgroSciences LLC conducted a groundwater monitoring study on a golf course near Orlando, FL, where a 1,3‐D‐containing product used on golf course fairways and greens, had been applied for six consecutive years at a rate of 55 kg ha^−1^. Groundwater was < 0.3 m deep for most of the study and at times was near the soil surface as a result of significant rainfall events. A network of eight monitoring wells, screened from 0.3 to 2.1 m below the ground surface, were installed 30.5 m directly down‐gradient from the 1,3‐D treated fairways. The soil at the site was predominantly Satellite and Immokalee series sand, with a hydraulic conductivity ranging from 0.3 m to 9 m per day as estimated by three separate rising‐head slug tests in each of the monitoring wells. The predicted groundwater velocity was 0.03–0.06 m day^−1^ and thus the site was extremely vulnerable to the potential leaching and transport of 1,3‐D and the 3‐chloroallyl alcohol and 3‐chloroacrylic acid metabolites. All wells were sampled once every 3 months for over 2 years (nine sampling events) from 2007 to 2009. Despite the sandy soil, shallow wells, high rainfall at the site, and six consecutive annual applications of 1,3‐D no 1,3‐D or the 3‐chloroallyl alcohol metabolite were detected (LOD = 0.02 μg L^−1^). A single detection of the 3‐chloroacrylic acid metabolite was observed at a level of 0.1 μg L^−1^ in one well.

### 3.5 Canadian 1,3‐D groundwater monitoring studies

#### 3.5.1 *Abbotsford aquifer (British Columbia)*

Grove *et al.* 18 conducted a groundwater monitoring study in an area of significant 1,3‐D use in the Abbotsford aquifer raspberry‐growing region of British Columbia, and sampled 35 wells for 1,3‐D on a monthly basis for 3 years (June 1991–June 1994). The wells were located in areas of the Abbotsford aquifer with high nitrate concentrations, and thus were suspected to be at most risk to the leaching of pesticides. The primary land use in the monitored area was raspberry production, which commonly required a high 1,3‐D application rate (275 kg ha^−1^) for nematode control. That study found 1,3‐D in a single monitoring well for five sampling periods (∼ 1300 samples) between December 1991 and March 1992, for a detection rate of 0.4%. The maximum observed concentration of 1,3‐D was 0.23 μg L^−1^, which quickly dissipated to non‐detectable levels (LOD = 0.1 μg L^−1^) in the next month's sample. The authors concluded that the paucity of 1,3‐D detections in this vulnerable scenario is not unexpected because the *cis*‐ and *trans*‐isomers of 1,3‐D are highly volatile and in wet soil are hydrolyzed to their corresponding 3‐chloroallyl alcohol. This study corroborates the results of extensive groundwater monitoring in the USA, but under Canadian climatic and edaphic conditions.

#### 3.5.2 *Southern Ontario tobacco growing region*

Merriman *et al.* 19 monitored for 1,3‐D in three groundwater monitoring wells, and four surface water locations in the Big Creek Watershed which drains a section of Norfolk County, Ontario, Canada, that had significant tobacco production and 1,3‐D use as a soil fumigant. The soils in the monitoring region consisted of well‐drained sands and shallow unconfined aquifers, which are typical of the southern Ontario tobacco‐growing region. Four monitoring wells were sampled from shallow groundwater in areas where 1,3‐D applications were made. Samples were collected during the typical spring application season for tobacco (April–May) and continued through one growing season. 1,3‐D was not detected in any of the groundwater samples.

### 3.6 Dow AgroSciences European Union groundwater monitoring studies

A 1,3‐D groundwater monitoring study was conducted as part of the 1,3‐D stewardship program in Europe. Groundwater samples from aquifers in close proximity to areas of high 1,3‐D use were taken in alignment with the Industry and Governmental Sustainable Agriculture policy. The monitoring program was conducted in partnership with academic and commercial organizations in the respective countries involved, including the University of Almeria (Spain), University Cattolica del Sacro Cuore Piacenza (Italy), ADAS UK Ltd and CEM Analytical Services (UK), National Agricultural Research Foundation (Greece) and ANTEA group (France). The program included five countries of major 1,3‐D use (Italy, Spain, France, UK, and Greece). Within each of these countries, five regions (four in Greece) of high 1,3‐D use were identified using authenticated regional sales information, photographic evidence of use, written statements confirming historical use of 1,3‐D in the region from local distributors. The relevant fumigated crops were assessed along with local hydrogeological characteristics of the region including depth of underlying aquifers, hydrological properties of the unsaturated zone beneath the plow layer and the aquifer, and the depth of groundwater accessed in potential sampling wells. This allowed targeting of areas that were known to be potentially vulnerable to the leaching of 1,3‐D and its metabolites, such as sandstone aquifers overlain by sandy soils and particularly where shallow water tables were present. The monitoring program design is described in detail by Terry *et al.* 13

Residues of 1,3‐D, 3‐chloroallyl alcohol or 3‐chloroacrylic acid exceeding *>*0.1 μg L^−1^ were not found in groundwater sampled in the UK, France, Italy and Greece from 2001 to 2004. The samples analyzed from Spain (∼ 1200) during the same period showed no residues of 1,3‐D or 3‐chloroallyl alcohol > 0.1 μg L^−1^. 3‐Chloroacrylic acid was found at *>* 0.1 μg L^−1^ in two different well the Caceres region (Spain) in March 2004 (0.12 μg L^−1^) and in November 2004 (0.4 μgL^−1^). These detections in Spain triggered monthly sampling at each well; however, no additional 1,3‐D residues were detected.^13^

Additional monitoring was conducted at the wells in UK, France, Italy and Spain between 2007 and 2009 resulting in 96 wells sampled and 562 samples analyzed (Table 3). That program showed three quantifiable detections of 1,3‐D ranging from 0.18 to 2.72 μg L^−1^.

**Table 3:** EU groundwater monitoring results from 2001 to 2016

Study region	Years	No. of wells	No. of samples	No. of detections^a^
United Kingdom	2001–2003	23	184	0
France	2001–2003	23	184	0
Italy	2002–2004	25	200	0
Spain	2002–2004	25	200	2^b^
Greece	2006‐2009	17	136	0
United Kingdom	2007–2009	23	167	0
France	2007–2009	23	126	2^c^
Italy	2007–2009	25	131	0
Spain	2007–2009	25	138	1^d^
Italy	2014–2016	10	81	2^e^
Spain	2014‐2016	10	85	3^f^

LOD, limit of detection; LOQ, limit of quantification.

a LOD = 0.02 μg L^−1^, LOQ = 0.05 μg L^−1^.

b 3‐Chloroacrylic acid, 0.12 and 0.4 μg L^−1^.

c 1,3‐D, 2.72 and 0.11 μg L^−1^.

d 1,3‐D, 0.18 μg L^−1^.

e 1,3‐D, 0.06 and 0.17 μg L^−1^.

f 1,3‐D, 0.73 and 0.48 μg L^−1^, 3‐chloroacrylic acid < LOQ.

Further monitoring was initiated in 2013 in high 1,3‐D use areas of Italy and Spain. Sites of recent 1,3‐D use (under the European Commission for Emergency Use Regulation SANCO/10087/2013) were mapped in relation to the ten selected monitoring wells in each of the high‐use areas in Italy and Spain. Monitoring resulted in 166 samples collected from 20 wells between 2014 and 2016, and showed five detections of 1,3‐D ranging from 0.06 to 0.48 μg L^−1^ (Table 3).

In total, from 2001 to 2014, a total of 115 unique groundwater wells in high 1,3‐D use areas in Europe were monitored for 1,3‐D and the 3‐chloroacrylic acid and 3‐chloroallyl alcohol metabolites, resulting in 1533 analyzed samples. Table 3 shows that 99.5% of the samples analyzed were < LOD, whereas seven quantifiable detections (i.e. > 0.05 μg L^−1^) of 1,3‐D and two quantifiable detections of 3‐chloroacrylic acid were found.

Evaluation of all of the EU groundwater monitoring data from 2001 to 2014 creates a large database of information from groundwater wells in vulnerable high‐use areas for 1,3‐D products in Europe. The extensive measured data, collected in areas where 1,3‐D had been used since the 1950s, show extremely low detection rates for 1,3‐D and its 3‐chloroallyl alcohol and 3‐chloroacrylic acid metabolites, and if detected, concentrations are extremely low. This is consistent with decades of groundwater monitoring data obtained from 1,3‐D high use areas in the USA and Canada, discussed above, which also show a very low frequency of trace level detections of 1,3‐D and its metabolites.

## 4 DISCUSSION

The lack of significant detections of 1,3‐D and its metabolites in groundwater after decades of study is the result of the high volatility of 1,3‐D, rapid degradation of 1,3‐D in most agricultural soils, rapid degradation of 1,3‐D and its metabolites in aerobic aquatic systems, and rapid hydrolysis of 1,3‐D in water. Potential leaching of 1,3‐D into groundwater is further mitigated by extensive label requirements that protect water, including engineering controls for minimizing end‐row spillage, shut‐off devices on all hoses and dry‐disconnect points to minimize leakage. Specific, mandatory label requirements in the USA are further protective of water resources: “do not apply directly to water, to areas where surface water is present, to intertidal areas below the mean highwater mark, within 100 feet of any well used for potable water, within 100 feet from the edge of karst topographical features.” In specific cold‐climate states in the USA (ND, SD, WI, MN, NY, ME, NH, VT, MA, UT, and MT), applications are prohibited to hydrologic group A soils where groundwater aquifers exist at a depth of 50 ft (15.2 m) or less from the surface.

The monitoring data summarized in this review further supports the US EPA OW determination to not regulate 1,3‐D with a National Primary Drinking Water Regulation (NPDWR). Furthermore, it corroborates the EPA's OW statement that “EPA believes the 1999 pesticide labeling requirements, which are intended to mitigate risks to drinking water, may be one reason for the infrequent occurrence of 1,3‐D at levels of concern in subsequent monitoring surveys.”^20^ and supports the OW analysis and conclusion that 1,3‐D appears to occur infrequently at levels of health concern in public water supplies. The data reviewed in this document support the OW conclusion that regulation of 1,3‐D with a NPDWR is not warranted, and furthermore that an NPDWR does not present a meaningful opportunity for health risk reduction for 1,3‐D.

The significant volume of recent and high‐quality groundwater monitoring data suggest that the mitigations imposed on the current Telone label restricting applications to > 100 ft (30.5 m)from potable wells, and restricting use in cold climate states (in the USA) with hydrologic group A soils and groundwater < 50 ft (15.2 m) deep, are effective in minimizing impact of 1,3‐D on groundwater resources.

## 5 CONCLUSIONS

Extensive groundwater monitoring data exist within the 1,3‐D use regions of the USA, Canada and the EU. This gives a historical review of exposure from over 50 years of product use within these regions. Since 1980, hundreds of groundwater or drinking water wells and tens of thousands of water samples have been analyzed for 1,3‐D, and more than 4500 samples have been analyzed for the 3‐chloroacrylic acid and 3‐chloroallyl alcohol metabolites. The groundwater monitoring studies reviewed here were conducted in areas of high 1,3‐D use and hydrogeologically vulnerable regions (i.e. shallow unconfined groundwater, sandy soils), and showed a very low rate of trace level detections of 1,3‐D or its 3‐chloroacrylic acid and 3‐chloroallyl alcohol metabolites. These data support the use of 1,3‐D soil fumigation products under “real‐world” conditions well within standards of acceptable exposure and risk, and corroborate the EPA OW conclusion that regulating 1,3‐D with a NPDWR is not warranted and further would not represent a meaningful opportunity for health risk reduction.
